# The FANTOM5 collection, a data series underpinning mammalian transcriptome atlases in diverse cell types

**DOI:** 10.1038/sdata.2017.113

**Published:** 2017-08-29

**Authors:** Hideya Kawaji, Takeya Kasukawa, Alistair Forrest, Piero Carninci, Yoshihide Hayashizaki

**Affiliations:** 1 RIKEN Preventive Medicine and Diagnosis Innovation Program, Wako, Saitama 351-0198, Japan; 2 RIKEN Advanced Center for Computing and Communication, Preventive Medicine and Applied Genomics Unit, Yokohama, Kanagawa 230-0045, Japan; 3 RIKEN Center for Life Science Technologies, Division of Genomic Technologies, Yokohama City, Kanagawa 230-0045, Japan; 4 RIKEN Omics Science Center, Yokohama City, Kanagawa 230-0045, Japan; 5 Harry Perkins Institute of Medical Research, Nedlands 6009, Western Australia, Australia

**Keywords:** Computational biology and bioinformatics, Systems biology, Molecular biology

## Abstract

The latest project from the FANTOM consortium, an international collaborative effort initiated by RIKEN, generated atlases of transcriptomes, in particular promoters, transcribed enhancers, and long-noncoding RNAs, across a diverse set of mammalian cell types. Here, we introduce the FANTOM5 collection, bringing together data descriptors, articles and analyses of FANTOM5 data published across the Nature Research journals. Associated data are openly available for reuse by all.

## Comment

Our genomes contain the complete set of information necessary to specify our development from a single totipotent cell to a complex multicellular organism, composed of hundreds of specialized cell types able to respond to environmental changes. In each of these cell types, and their responding states, different sets of genes are expressed through transcription. Determining the transcriptome, including the set of genes expressed, is fundamental to understanding cellular identity, gene regulation and human disease. The FANTOM (Functional Annotation of Mammalian Genomes) project was launched to provide a comprehensive catalogue of transcripts encoded in mammalian genomes (http://fantom.gsc.riken.jp). With the full-length cDNA technology developed at RIKEN^1^, the first, second and third rounds of the FANTOM projects surveyed the mammalian transcriptome landscape by sequencing a large collection of full-length cDNAs. This improved our catalog of protein coding genes, but also revealed the new world of long non-protein coding RNAs^2–7^ (a major novel class of genes that had been overlooked). The cap-trapper reaction, initially used to select full-length cDNAs, was later used to develop CAGE (Cap Analysis Gene Expression) that quantifies transcription starting sites (TSSs) at single base-pair resolution^8^. With this method, the FANTOM3 project globally mapped TSSs in the mouse genome. This helped classify mammalian promoters into broad-CpG and sharp-TATA associated promoter architectures^9^. Subsequently the FANTOM4 project used CAGE and predicted proximal promoter transcription factor binding motifs to decipher the transcriptional regulatory network of a myeloid leukemia cell line undergoing differentiation^10^. Additionally, the new CAGE data revealed that a large fraction of the transcriptome initiates from retrotransposon derived sequences, and these exhibit exquisite tissue specificity^6^.

Most recently the FANTOM5^11–13^ project aimed at comprehensive maps of transcription initiation activities across the most diverse collection of cell types studied to date. A focus on normal, primary cells differentiated FANTOM5 from previous transcriptome studies. Most other broad studies had focused on tissues (heterogeneous mixtures of cell types) or cancer cell lines (atypical cell states). The key technology developed for the project was a variation of CAGE adapted to a single molecule sequencer, HeliScope^14^. An advantage of this variation of CAGE was the reduction of the required input material down to 100 ng of total RNA^15^, approximately 100 fold lower than the amount required in FANTOM4^10^. The reduced sample requirements allowed us to profile rarer cell populations and thus cover a broader range of cell types. The other advantage of single molecule sequencing was improved accuracy of quantification. Single molecule sequencing avoided PCR induced amplification biases seen with other sequencers. We note that although the HeliScope is no longer commercially available, single molecule CAGE libraries can still be sequenced at SeqLL^16^ using a related technology.

More than three thousand human and mouse samples were collected and profiled in FANTOM5. The main focus was on mapping TSSs using single molecule CAGE, however for a subset of these samples we also applied RNA-seq, and small RNA-seq to study long-noncoding RNAs^17^ and microRNA promoters^18^, and CAGEscan^19^ (another variation of CAGE) to link promoters to downstream exons. Additionally we profiled a smaller number of samples from rat, dog, chicken and macaque to study TSS orthology and turnover (Table 1).

The immediate outcome of the CAGE data was the promoter-level expression atlas consisting of approximately 201,000 and 158,000 CAGE peaks in 1,900 human and 1,200 mouse samples, respectively^11,13^. In-depth examination of the CAGE signal also allowed identification of 65,000 and 44,000 enhancers in the human and mouse genome based on the eRNA (enhancer RNA) expression profiles^12,13^. Its integration with RNA-seq in human identified ~28,000 long non-coding RNAs with high-confidence 5′-ends^17^. With the realization that we could quantify activities of both promoters and enhancers we used CAGE to monitor multiple time-series or differentiation and response which revealed that transcribed enhancers lead waves of coordinated transcription^13^. In addition to the mapping of genomic features the expression atlas has been used to select key transcription factors for trans differentiation experiments, identify novel biomarkers, and uncover molecular basis in a wide range of context. Data underlying the atlas have been compiled into the FANTOM5 web resource^20^ and also integrated with complementary resources. The data are open and being broadly used outside of the consortium, where the articles on the promoter- and the enhancer-atlas^11,12^ are heavily cited.

The FANTOM5 data can be used more broadly. In order to facilitate data use from wider aspects, this collection aims to provide a data-centric perspective of the FANTOM5 project with Data Descriptors of individual datasets. The collection consists of published data, previously unpublished data, reprocessed data and meta-analyses. We launch this collection with a limited number of articles, but it will grow until the entire data set is published. We believe that the articles in this collection (www.nature.com/collections/fantom5) coupled with metadata records curated by *Scientific Data* will stimulate the use of our data in many areas of life sciences.

## Additional Information

**How to cite this article:** Kawaji, H. *et al.* The FANTOM5 collection, a data series underpinning mammalian transcriptome atlases in diverse cell types. *Sci. Data* 4:170113 doi: 10.1038/sdata.2017.113 (2017).

## Figures and Tables

**Table 1:** Transcriptome profiles obtained in the FANTOM5 project.

	**CAGE with a single molecule sequencer**	**CAGE scan**	**RNA-seq**	**Small RNA-seq**	**Total**
Human (*Homo sapiens*)	1,885	124	70	423	2,570
Mouse (*Mus musculus*)	1,202	—	—	78	1,280
Rat (*Rattus norvegicus*)	13	—	—	6	19
Dog (*Canis lupus familiaris*)	13	—	—	6	19
Chicken (*Gallus gallus*)	32	—	—	5	37
Rhesus (*Macaca mulatta*)	15	—	—	—	15
Total	3,228	124	70	518	3,940
